# Medical Students' Competency in EFAST (Extended Focused Assessment Sonography for Trauma) Components: A Prospective Observational Study

**DOI:** 10.7759/cureus.69338

**Published:** 2024-09-13

**Authors:** Munawar Farooq, Arif Alper Cevik, David O Alao, Virgie Guy Pedo, Fikri Abu-Zidan

**Affiliations:** 1 Department of Internal Medicine, Emergency Medicine Section, College of Medicine and Health Sciences, United Arab Emirates University, Al Ain, ARE; 2 Department of Emergency Medicine, Tawam Hospital, Al Ain, ARE; 3 Department of Internal Medicine, College of Medicine and Health Sciences, United Arab Emirates University, Al Ain, ARE; 4 Research Office, College of Medicine and Health Sciences, United Arab Emirates University, Al Ain, ARE

**Keywords:** efast, emergency medicine, emergency ultrasound, medical student, skill, trauma

## Abstract

Objective: Ultrasound training in undergraduate medical education is developing, and its incorporation into the curriculum requires careful planning. Extended Focused Assessment Sonography for Trauma (EFAST) is commonly taught to medical students as one of the primary applications of ultrasound. Because false-negative EFAST scans can affect patients' clinical outcomes, it is essential to evaluate the individual components of this skill. We aim to determine which EFAST components students perform sub-optimally after initial training.

Methods: In this prospective observational study, 90 medical students from two final-year cohorts were assessed in EFAST components after uniform training during the emergency medicine clerkship. All validated components of the standard EFAST exam were assessed, and a descriptive analysis of individual components of EFAST was performed.

Results: The hepatorenal space, splenorenal space, and pelvic space fluid investigations had the lowest completion rates. Pericardial fluid, pelvic free fluid, and right thoracic pleural fluid investigations were often incorrectly applied. Fanning was most commonly missed in hepatorenal, splenorenal, and pelvic free fluid investigations, and between 12% and 50% of EFAST components had omitted reporting.

Conclusions: There were significant incomplete assessments for free intraperitoneal fluid, primarily due to a lack of fanning in the hepatorenal, splenorenal, and pelvic areas. Trainers can effectively enhance student performance and outcomes by targeting these challenging areas. Further research might reveal whether residents and physicians show similar trends in EFAST completion.

## Introduction

Point-of-care ultrasound (POCUS) uses handheld or portable ultrasound at the patient’s bedside [1]. It has become a clinical tool for many doctors in different specialties. It answers focused clinical questions for refining diagnostic and management decisions [2]. Advances in POCUS technology are making it accessible in different clinical settings. In 2012, the Accreditation Council for Graduate Medical Education and the American Board of Emergency Medicine included ultrasound as a milestone for emergency medicine residents [3]. Incorporating ultrasound training at the residency and fellowship levels suggests that trainees would benefit from earlier exposure in medical schools [4]. Ultrasound education is well-established in some undergraduate medical institutions, while many have yet to start. Incorporating ultrasound into undergraduate medical education is feasible and beneficial, but barriers include a cramped curriculum and a lack of capital and trained faculty [5,6]. A World Ultrasound Society consensus statement concluded that ultrasound training in undergraduate medical education is still developing and requires careful planning for proper incorporation [7].

Extended Focused Assessment with Sonography for Trauma (EFAST) is the application of ultrasound in clinical decision-making. It is a common protocol for teaching ultrasound in postgraduate education [8-10]. As one of the most common bedside ultrasound procedures practiced in emergency departments, many medical schools worldwide have included EFAST in their emergency medicine clerkship (EMC) curriculum [11,12]. Gogalniceanu et al. and Cevik et al. reported the feasibility of training medical students in FAST and EFAST scanning within short time frames, 5 and 4 hours, respectively [13,14].

EFAST has a wide range of sensitivity and specificity [15,16]. Sensitivity and specificity were found to be lower in trainees [17]. A recent systematic review reported that the sensitivity of EFAST is generally lower than its specificity [18]. Moreover, the reported values differ for the individual components of EFAST. The sensitivity for pericardial fluid was 91%, for intraabdominal free fluid was 74%, and for pneumothorax was 69%. In comparison, the specificities for the different components ranged from 94% to 99% [18]. There is limited literature on trainees' performance in the different components of EFAST scans. We aimed to define the components of EFAST that medical students perform poorly after initial training. Trainers can effectively enhance student performance and outcomes by targeting these challenging areas.

## Materials and methods

Methods

Study Design and Setting

This prospective observational study was conducted at a medical college in the United Arab Emirates (UAE) with a six-year undergraduate medical curriculum. EMC is a four-week rotation for final-year medical students [19]. One of the clinical skills taught in the EMC curriculum is the EFAST protocol. The manuscript analyzes prospective EFAST assessment data collected over two years.

Participants

The participants were final-year medical students tested on EFAST stations in their Objective Structured Clinical Examination (OSCE) during the 2016-17 and 2017-18 academic years. Before this clerkship, the participants had no prior exposure to ultrasound training.

Sample Size

The study included records of all 90 final-year medical students who participated in the EFAST stations of their OSCE during the two academic years.

Materials and measurement

Ultrasound Equipment

Training and testing were performed with a Siemens Acuson P300 ultrasound machine with a low-frequency (2-5 MHz) convex transducer and a high-frequency (8-18 MHz) linear transducer.

Teaching Sessions

During the 2016-17 and 2017-18 academic years, a single tutor with 15 years of experience in POCUS taught all final-year medical students how to perform EFAST on simulated patients in the clinical skills lab. The students received a 1-hour didactic lecture on basic physics, knobology, artifacts, and the EFAST technique, followed by a 2-hour practical session on human models under the tutor’s supervision. Three intraperitoneal (hepatorenal space, splenorenal space, pelvic space) and four intrathoracic lung views (right and left pneumothorax investigation, right and left intrathoracic free fluid investigation) and one subcostal pericardial view were used in EFAST training. Students were instructed on the application, normal, and pathological views during the didactic session. During the practical session, they learned how to achieve an acceptable view for each sonographic window. The didactic and practical sessions provided anatomy landmarks, views, fanning, tilting, and reporting details. During their clerkship, students were required to perform at least three EFAST assessments on trauma patients in the Emergency Department under the direct supervision of an attending emergency physician. During the final week of rotation before the OSCE, the students received an additional 2-hour free practice on human models.

Assessment

The same tutor who conducted didactic and practical sessions evaluated the students' EFAST performance on simulated patients in a station as part of the final clerkship OSCE. The assessment form included all validated components of the standard EFAST exam [14]. The EMC OSCE included 11 stations (eight active stations) for 6 minutes each with no modifications throughout the two-year study period.

Outcome Measures

The Electronic Objective Structured Clinical Evaluation (eOSCE) desktop program was used to create OSCE evaluation sheets [20]. Data for each student's performance was collected in a cloud-based platform, and the results were downloaded as an Excel spreadsheet. On the marking sheet, there were 12 components. The EFAST exam components listed in the marking sheet (Table 1) included two categories: preparation and investigation. The preparation section had four components coded as completed, not attempted, or wrong selection/application. The investigation section had eight components coded as completed, partially completed, inadequately done, not attempted, and wrong application. "Completed" was checked if all required items in an EFAST component were achieved. "Partially completed" was checked if more than half but not all items were achieved. "Inadequately done" was checked if less than half of the items were achieved. Other options on the form were “not attempted” for missed components and “wrong application” if an incorrect examination was applied in that particular EFAST component. In the comments section of the marking sheet, the examiner wrote important areas that the student overlooked or had done wrongly.

**Table 1:** Components of Extended Focused Assessment With Sonography for Trauma in Assessment Sheet

Preparation
1. Introduce him/herself to the patient appropriately and explain the procedure.
Completed/Not Attempted
2. Warn the patient for cold gel.
Completed/Not Attempted
3. Choose the right transducer to start the exam.
Completed/Wrong Selection
4. Place the transducer marker to correct direction.
Completed/Wrong Application
Investigation
1. Pericardial fluid (correctly show subcostal pericardial view, use transthoracic parasternal long-axis view as backup, report)
Completed/Partially Completed/Inadequately Done/Not Attempted/Wrong Application
2. Intraperitoneal fluid - hepatorenal space (place the transducer at the midaxillary - costal margin line point, show the Morrison pouch, apply fanning to investigate all areas of Morrison pouch, report)
Completed/Partially Completed/Inadequately Done/Not Attempted/Wrong Application
3. Right thoracic pleural fluid (hemothorax) (place the transducer at the midaxillary point, show the diaphragm, show lung, diaphragm, and liver view, report)
Completed/Partially Completed/Inadequately Done/Not Attempted/Wrong Application
4. Intraperitoneal fluid - splenorenal space (place the transducer at the posterior axillary-xiphoid line point, show the splenorenal space, apply fanning to investigate all areas of splenorenal space, report)
Completed/Partially Completed/Inadequately Done/Not Attempted/Wrong Application
5. Left thoracic pleural fluid (hemothorax) (place the transducer at the posterior axillary line, show the diaphragm, show lung, diaphragm, and spleen view, report)
Completed/Partially Completed/Inadequately Done/Not Attempted/Wrong Application
6. Pelvic free fluid (correctly apply transverse view above symphysis pubis, apply fanning in transverse view and show all areas, correctly apply sagittal view above symphysis pubis, apply fanning in sagittal view and show all areas, report)
Completed/Partially Completed/Inadequately Done/Not Attempted/Wrong Application
7. Right Pneumothorax (place the transducer at the midclavicular and second third intercostal point, show two ribs and pleural movements on screen, correctly apply M-mode, report)
Completed/Partially Completed/Inadequately Done/Not Attempted/Wrong Application
8. Left pneumothorax (place the transducer at the midclavicular, second, and third intercostal point, show two ribs and pleural movements on screen, correctly apply M-mode, report)
Completed/Partially Completed/Inadequately Done/Not Attempted/Wrong Application

Analysis

Descriptive analyses were performed to address common missing steps for each EFAST component. Examiner notes were reviewed to address common mistakes or issues. Nonparametric statistical methods were used. Fisher's exact test was used to determine the significance of the association between two categorical variables. Mann-Whitney U tests were used for continuous variables. Values are presented as medians and ranges or numbers and percentages where applicable. Statistical significance was defined as a probability less than 0.05. The Statistical Package for the Social Sciences was used to analyze the data (IBM-SPSS version 28, Chicago, IL).

Ethical approval for this study was obtained from the Research and Graduate Studies Ethics Committee under approval number ERS_2017_5643, dated 19th September 2017. Given that the study involved the use of anonymized educational data from a mandatory component of students’ training and assessment, the ethics committee granted an exemption from obtaining informed consent from individual students.

This study adheres to the STROBE (Strengthening the Reporting of Observational Studies in Epidemiology) guidelines for reporting observational studies.

## Results

There were five student groups in each academic year and a total of 146 students (66 in 2016-17 and 80 in 2017-18). Six out of the 10 student groups (90 students, 62%) received an EFAST station in their clerkship OSCE. Twenty-six students (two groups) were in the 2016-17 academic year, and 64 students (four groups) were in the 2017-18 academic year. Seventy percent of the students (63) were female. There were no statistically significant differences between the students in the two academic years regarding gender distribution, final EM marks, final OSCE marks, EFAST station marks, or OSCE marks (without EFAST station) (Table 2).

**Table 2:** Students and their emergency medicine marks in two academic years Fisher's exact and Mann-Whitney U tests were used to calculate p-values. Nominal values were presented as numbers and percentages. Continuous numbers were presented as median and range. EM: Emergency Medicine, OSCE: Objective Structured Clinical Examination, EFAST: Extended Focused Assessment With Sonography for Trauma.

	2016-17 (n = 26)	2017-18 (n = 64)	p-Value
Gender			0.31
Female	16 (61.5%)	47 (73.4%)	
Male	10 (38.5%)	17 (26.6%)	
EM Final Mark	84.5 (78.0-89.0)	84.5 (67.0-90.0)	0.82
EM OSCE Mark	80.8 (71.8-85.9)	80.0 (67.8-89.8)	0.38
EFAST Station Mark	75.0 (45.8-91.7)	70.8 (33.3-100)	0.93
EM OSCE Mark (Without EFAST Station)	81.6 (72.4-87.0)	80.7 (67.7-90.7)	0.32

Table 3 shows the components of the EFAST marking sheet and student performances. Students' most successfully completed EFAST components were pericardial fluid, right chest pneumothorax, and left chest pneumothorax investigations (78.9%, 66.7%, and 63.3%, respectively). The three components in the abdominopelvic investigation section had the lowest “completed” marks: intraperitoneal fluid - hepatorenal space (31.1%), intraperitoneal fluid - splenorenal space (31.1%), and pelvic free fluid (28.9%). The top three components marked as "wrong application" in the investigation section were pericardial fluid (8.9%), right thoracic pleural fluid (6.7%), and pelvic free fluid (6.7%) investigations.

**Table 3:** EFAST components and students’ performances OSCE: Objective Structured Clinical Examination; EFAST: Extended Focused Assessment With Sonography for Trauma; N/A; not available/applicable.

Steps of OSCE in Marking Sheet	Completed	Partially Completed	Inadequately Done	Not Attempted	Wrong Application
	N (%)	N (%)	N (%)	N (%)	N (%)
Preparation					
Introduce him/herself to the patient appropriately and explain the procedure	73 (81.1)	N/A	N/A	14 (18.8)	N/A
Warn the patient for cold gel	43 (47.8)	N/A	N/A	47 (52.2)	N/A
Choose the right transducer to start the exam	89 (98.9)	N/A	N/A	N/A	1 (1.1)
Place the transducer marker in the correct direction	83 (92.2)	N/A	N/A	N/A	7 (7.8)
Investigation					
Cardiac investigation					
Pericardial fluid	71 (78.9)	11 (12.2)	-	-	8 (8.9)
Abdominopelvic investigation					
Intraperitoneal fluid - hepatorenal space	28 (31.1)	57 (63.3)	3 (3.3)	-	2 (2.2)
Intraperitoneal fluid - splenorenal space	28 (31.1)	55 (64.4)	3 (3.3)	-	4 (4.4)
Pelvic free fluid	26 (28.9)	41 (45.6)	16 (17.8)	1 (1.1)	6 (6.7)
Thoracic investigation					
Right thoracic pleural fluid (hemothorax)	31 (34.4)	34 (37.8)	12 (13.3)	7 (7.8)	6 (6.7)
Left thoracic pleural fluid (hemothorax)	33 (36.7)	34 (37.8)	6 (6.7)	13 (14.4)	4 (4.4)
Right pneumothorax	60 (66.7)	14 (15.6)	6 (6.7)	8 (8.9)	2 (2.2)
Left pneumothorax	57 (63.3)	10 (11.1)	4 (4.4)	15 (16.7)	4 (4.4)

Table 4 shows the missed items in each EFAST investigation component.

**Table 4:** Missed items by students in each EFAST investigation component. OSCE: Objective Structured Clinical Examination; EFAST: Extended Focused Assessment With Sonography for Trauma.

Components and Items of EFAST in OSCE Marking Sheet	Yes N (%)	No N (%)
Cardiac investigation		
Pericardial fluid		
Correctly show subcostal pericardial view	69 (76.7)	21 (23.3)
Use transthoracic parasternal long-axis view as backup (n = 21)	12 (57.1)	9 (42.9)
Report	79 (87.8)	11 (12.2)
Abdominopelvic investigation		
Intraperitoneal fluid - hepatorenal space		
Place the transducer at the mid-axillary-costal margin line point	86 (95.6)	4 (4.4)
Show the Morrison pouch	80 (88.9)	10 (11.1)
Apply fanning to investigate all areas of the Morrison pouch	38 (42.2)	52 (57.8)
Report	70 (77.8)	20(22.2)
Intraperitoneal fluid - splenorenal space		
Place the transducer at the posterior axillary-xiphoid line point	81 (90.0)	9 (10.0)
Show the splenorenal space	82 (91.1)	8 (8.9)
Apply fanning to investigate all areas of the splenorenal space	38 (42.2)	52 (57.8)
Report	63 (70.0)	27 (30.0)
Pelvic free fluid investigation		
Correctly apply transverse view above the symphysis pubis	81 (90.0)	9 (10.0)
Apply fanning in transverse view and show all areas	45 (50.0)	45 (50.0)
Correctly apply sagittal view above the symphysis pubis	73 (81.1)	17 (18.9)
Apply fanning in sagittal view and show all areas	41 (45.6)	49 (54.4)
Report	66 (73.3)	24 (26.7)
Thoracic investigation		
Left thoracic pleural fluid (hemothorax)		
Place the transducer at the posterior axillary line	59 (65.6)	31 (34.4)
Show the diaphragm	67 (74.4)	23 (25.6)
Show lung, diaphragm, and spleen view	56 (62.2)	34 (37.8)
Report	45 (50.0)	45 (50.0)
Right thoracic pleural fluid (hemothorax)		
Place the transducer at the mid-axillary point	60 (66.7)	30 (33.3)
Show the diaphragm	69 (76.7)	21 (23.3)
Show lung, diaphragm, and liver view	53 (58.9)	37 (41.1)
Report	45 (50.0)	45 (50.0)
Right pneumothorax		
Place the transducer at the midclavicular, second, and third intercostal point	76 (84.4)	14 (15.6)
Show two ribs and pleural movements on the screen	74 (82.2)	16 (17.8)
Correctly apply M-mode	68 (75.6)	22 (24.4)
Report	69 (76.7)	21 (23.3)
Left pneumothorax		
Place the transducer at the midclavicular, second, and third intercostal point	66 (73.3)	24 (26.7)
Show two ribs and pleural movements on the screen	64 (71.1)	26 (28.9)
Correctly apply M-mode	66 (73.3)	24 (26.7)
Report	64 (71.1)	26 (28.9)

Most students could find anatomical structures and show appropriate views of each component. However, items in each component were frequently missed. Students lacked the skill to apply fanning in hepatorenal, splenorenal, and pelvic windows. Lack of reporting of findings was the main item missed in thoracic pleural fluid investigations.

## Discussion

The hepatorenal, splenorenal, and pelvic space-free fluid investigations had the lowest full completion rates. Pericardial fluid, pelvic free fluid, and right thoracic pleural fluid investigations were frequently marked as wrong applications. Fanning was most commonly overlooked in hepatorenal, splenorenal, and pelvic free fluid investigations. Additionally, there was a lack of reporting in the investigations of thoracic pleural fluid.

No other studies in the literature have specifically reported the completion rate of individual components of EFAST scans by trainees. Since the frequency of incomplete scans will affect the accuracy of EFAST, the high number of partially completed scans for intraperitoneal fluid suggests an increased risk of false-negative scans. Our study found that over 60% of scans in the hepatorenal and splenorenal areas were incomplete. This finding needs to be correlated with findings from clinical studies. A review of EFAST scans in trauma patients found that the most common cause of false-negative scans was missing free fluid in the pelvis, right hypochondrium, or left hypochondrium, with 31% of these false-negative scans necessitating operative or interventional radiology procedures [21]. This highlights the clinical relevance of conducting complete scans, including a proper fanning technique.

There is evidence that different ultrasound applications in emergency medicine differ in their learning curve. A study reported that the number of exams needed for soft tissue exams was only 18, while for good interpretation skills for right upper quadrant ultrasound examination, the number of scans needed was 90 [22]. Bleahr et al. also highlighted that EFAST needed 57 examinations to reach its highest interpretation plateau [22]. Acceptable interpretation comes with experience in using proper ultrasound techniques on a daily basis. Our students did not have the chance to examine a high number of patients during the four weeks of EMC. However, our study demonstrated that uniform training of EFAST results in variances in different components of EFAST during a short period of time. This suggests different learning curves for individual components of EFAST, which need to be realized by EFAST trainers. Tailoring training programs to address these differences is essential to ensure consistent and competent performance across all aspects of EFAST.

Incomplete documentation of EFAST scans in clinical retrospective studies was up to 30% [21]. In our study, the lack of reporting ranged from 12.2% in the pericardial window to 50% in the hepatorenal and splenorenal windows. These results reveal the prevalence of incomplete reports in EFAST scans, suggesting a need for more comprehensive practices to ensure the accuracy of EFAST assessments and reporting.

Limitations

This study has the following notable limitations. First, the whole process of training and assessment was completed within four weeks. The four-week duration may be too short for the effective acquisition and retention of competencies. Therefore, the findings might not apply to training conducted over longer periods of time, such as residency programs. Second, the study did not assess retention of the learned skills after six months or longer. Third, the assessment scans were conducted on simulated patients with easy-to-scan anatomy. These conditions may not apply to real-life trauma patients. Finally, only one trainer conducted both the training and the EFAST assessment of all the students. This may have affected the reliability of the assessment. Nevertheless, the long ultrasound teaching experience of the assessor may have reduced this effect.

## Conclusions

In summary, final-year medical students performed significant incomplete EFAST assessments for free intraperitoneal fluid, mostly due to a lack of fanning in the hepatorenal, splenorenal, and pelvic areas. This should be considered by educators and trainers, including senior physicians, in both undergraduate and postgraduate training programs. This finding has also been reported in clinical studies. Further studies on the assessment of EFAST competencies in residents and other physicians will indicate whether this pattern of incomplete scans and missed reporting exists after training programs in other training and clinical settings.
